# Prevalence and correlates of positive mental health in Chinese adolescents

**DOI:** 10.1186/s12889-018-5133-2

**Published:** 2018-02-17

**Authors:** Cheng Guo, Göran Tomson, Christina Keller, Fredrik Söderqvist

**Affiliations:** 1 Medical Management Centre, Department of Learning, Informatics, Management and Ethics, Karolinska Institutet, Stockholm, Sweden; 2 Department of Public Health Sciences, Karolinska Institutet, Stockholm, Sweden; 3 International Business School, Jönköping University, Jönköping, Sweden; 4 Center for Clinical Research, Uppsala University, Västerås Hospital, Västerås, Sweden; 5 Competence Center for Health, Region of Västmanland, Västerås Hospital, Västerås, Sweden

**Keywords:** Positive mental health, Chinese adolescents, Prevalence, Correlates, Mental Health Continuum-Short Form (MHC-SF)

## Abstract

**Background:**

Studies investigating the prevalence of positive mental health and its correlates are still scarce compared to the studies on mental disorders, although there is growing interest of assessing positive mental health in adolescents. So far, no other study examining the prevalence and determinants of positive mental health in Chinese adolescents has been found. The purpose of this study was to assess the prevalence and correlates of positive mental health in Chinese adolescents.

**Methods:**

This cross-sectional study used a questionnaire including Mental Health Continuum-Short Form (MHC-SF) and items regarding multiple aspects of adolescent life. The sample involved a total of 5399 students from grade 8 and 10 in Weifang, China. Multivariate Logistic regression analyses were performed to evaluate the associations between potential indicators regarding socio-economic situations, life style, social support and school life and positive mental health and calculate odds ratios and 95% confidence intervals.

**Results:**

More than half (57.4%) of the participants were diagnosed as flourishing. The correlated factors of positive mental health in regression models included gender, perceived family economy, the occurrence of sibling(s), satisfaction of self-appearance, physical activity, sleep quality, stress, social trust, desire to learn, support from teachers and parents as well as whether being bullied at school (OR ranging from 1.23 to 2.75). The Hosmer-Lemeshow *p*-value for the final regression model (0.45) indicated adequate model fit.

**Conclusion:**

This study gives the first overview on prevalence and correlates of positive mental health in Chinese adolescents. The prevalence of positive mental health in Chinese adolescents is higher than reported in most of the previous studies also using MHC-SF. Our findings suggest that adolescents with advantageous socio-economic situations, life style, social support and school life are experiencing better positive mental health than others.

## Background

Mental health is defined as a state of well-being in which every individual realizes his or her own potential, can cope with the normal stresses of life, can work productively and fruitfully, and can make a contribution to his or her community [1]. So far, there has been no global definition of positive mental health although many researchers have made attempts based on various theories [2, 3]. For example, Keyes suggested that mental health should be operationalized as a syndrome of symptoms of positive feelings and positive functioning in life [4]. The Public Health Agency of Canada referred to positive mental health as being ‘the capacity of each and all of us to feel, think, and act in ways that enhance our ability to enjoy life and deal with the challenges we face. It is a positive sense of emotional and spiritual well-being that respects the importance of culture, equity, social justice, interconnections and personal dignity’ [5]. The definitions of positive mental health are, and should be to some degree, context dependent [6]. Thus, Vaingankar et al. defined positive mental health as ‘the ability to build and maintain relationships, possess coping skills, pursue personal growth and autonomy, and participate in religious and spiritual practices’ in an Asian context [7]. Generally, the hedonic tradition dealing with positive emotions and the eudaimonic tradition focusing on optimal functioning of an individual in everyday life, dominate the field regarding the components of positive mental health [8]. The hedonic tradition refers to the desire to maximize pleasure and to minimize pain from the perspective of maximizing the good in one’s life [9]. Commonly contrasted with the hedonic tradition, the eudaimonic tradition includes virtue and effort as essential parts of happiness [10]. Some researchers underestimated the eudaimonic tradition in their model of positive mental health [10]. Considering these two ancient Greek approaches simultaneously, however, positive mental health can be defined as the presence of general emotional, psychological, and social well-being, as the concept adopted in this study [4]. Gains in positive mental health predict declines in mental disorders, while losses of positive mental health predicted increases in mental disorders [11]. However, this did not imply that mental health is the same as the absence of mental disorder. A growing body of evidence shows that high levels of well-being are good for individuals and society, and are associated with a range of positive outcomes, for example, good health and life expectancy and satisfaction [12]. As Merriam-Webster defines, well-being is the state of doing well especially in relation to one’s happiness or success [13]. This is related to the definition of positive mental health, as WHO defined mental health as state of well-being in a positive way [1].

Assessing the prevalence of positive mental health is challenging, partly because of the conceptually distinct definitions leading to various ways of operationalizing mental health [2]. One way developed over time is to use the short form of the Mental Health Continuum (MHC-SF), a scale developed by Keyes that measures three levels of positive mental health: flourishing, moderate and languishing mental health [14]. The instrument was developed based on the two-continua model that identifies mental health and mental illness as two related but distinguishable dimensions. Thus, the presence of mental health is not equal to the absence of mental illness. In this theory, positive mental health is emphasized as a combination of feeling good and functioning well in life [15]. Briefly, people who are flourishing in life report high levels of well-being, meaning that they often experience positive emotions and function well from both psychological and social perspectives. On the other hand, languishing is the absence of mental health as a state of being mentally unhealthy, equivalent to stagnation and emptiness or that life lacks interest and engagement [4]. The MHC-SF has been successfully tested on adults from South Africa, Poland, Italy, Brazil, United States and Australia, and adolescents from Egypt, India and South Korea [16–24] and has also shown good psychometric properties on a sample of Chinese adults [25]. The authors of this article evaluated the psychometric properties of the MHC-SF and found the instrument to be valid and reliable in assessing positive mental health also in Chinese adolescents [26]. Table 1 shows the percentages of flourishing individuals in different countries from previous studies. The lowest level of flourishing mental health was found in South Korean adolescents (11.7%), while the highest level was identified in Canadian adolescents and adults (76.9%).

**Table 1:** Prevalence of positive mental health assessed by MHC-SF in previous studies

Author(s), year	Target group	Age span	Sample size	Prevalence	Country
Lim, 2014 [16]	Adolescents	10th grade, mean 16.08	547	11.7%	South Korea
Karas et al., 2014 [17]	Aged above 16	16–81	2115	26.0%	Poland
Petrillo et al., 2014 [18]	Adults	18–89	1438	30.9%	Italy
Salama-Younes, 2011 [19]	Adolescents	12–18	339	23.48%	Egypt
Keyes et al., 2008 [20]	Adults	30–80 or older	1050	20%	South Africa
Keyes, 2005 [14]	Adults	25–74	3032	18%	United States
Dyrbye et al., 2012 [61]	College students	Not applicable	4400	53.1%	United States
Gilmour, 2014 [15]	Adolescents and adults	15–75 or older	25,113	76.9%	Canada
Yin et al., 2013 [25]	Adults	20–60 or older	2021	43.4%	China
Singh et al., 2015 [21]	Adolescents	13–18	539	46.4%	India

Multiple indicators of positive mental health in general populations have been identified across countries. Common indicators of positive mental health include socio-demographic factors [4, 15, 27–33], health status [15, 31], physical activity [32, 34–37], body image [38], sleeping [39], screen time [36, 40], substance use [32, 41, 42], social support [28, 30, 32, 33] and violence or discrimination [30]. For positive mental health of adolescents, school related factors such as peer relationship and support from teachers also play an important role [41]. Age was found to be associated with positive mental health in adverse directions under different contexts [4, 15, 28, 32]. Education, income, employment and living area were positively associated with positive mental health [15, 28–33]. Ethnicity also proved to be an indicator of positive mental health [27]. Socio-economic factors were found to be significantly associated with positive mental health in Chinese adults [25]. Other findings showed that physical activity was a significant predictor of positive mental health in Chinese college students [35]. Perceived discrimination was negatively linked to psychological well-being among Chinese migrant adolescents [43]. Furthermore, conceptual frameworks for the evaluation of positive mental health and its determinants have been developed. Orpana et al. defined 25 determinant indicators of positive mental health of children, youth and adults in Canada at the individual, family, community and societal level [5]. The individual indicators included physical activity, substance use, nurturing childhood environment, resilience, control and self-efficacy, spirituality, violence, and coping. Family indicators were household composition, family relationships, parenting style, family physical and mental health status, substance use among family members, and family income. Community indicators comprised social support, social network, school environment, workplace environment, community involvement, neighborhood social environment, and neighborhood built environment. Finally, the indicators at the societal level included inequality, discrimination, as well as political participation. According to Maher and Waters [44], indicators of positive mental health at the individual level for children generally refer to the presence of social connections and a strong sense of self and self-worth, and may include measures of a sense of belonging, self-esteem, engagement, self-determination and control and quality of life. Family indicators may include parental mental health, freedom from violence, family cohesion, parent-child attachment and use of responsive, developmentally-appropriate family and parenting practices. It is notable longitudinal research regarding positive mental health is still scarce [36, 39].

Researchers have focused on the evaluation of mental health using the measurement of mental disorders during the past decades [2]. To date, studies investigating the prevalence of positive mental health and its correlates are still scarce compared to the studies on mental disorders, although there is growing interest of assessing positive mental health in adolescents. Adolescence is a time in life that harbors many risks but also presents great opportunities for sustained health and wellbeing through education and preventive efforts [45]. There is mounting evidence that many, if not most, lifetime psychiatric disorders will first appear in childhood or adolescence [46]. Therefore, promoting mental health and identifying individuals at risk in adolescents is essential to reduce a heavy burden of disease worldwide. Positive mental health is not only believed to be inversely associated with mental disorders (but not two sides of the same continuum), especially in adolescence [11, 47], but also linked to positive outcomes in life [2]. In these longitudinal studies, researcher found that mental problems are predictive of declines in future positive mental health. There are knowledge gaps of prevalence, risks and protective factors of positive mental health as well as differences in positive mental health related to e.g. gender and socio-economic factors [48]. So far, no other study examining the prevalence and determinants of positive mental health in Chinese adolescents has been found. The purpose of this study was to analyze the prevalence of positive mental health and explore the correlates of positive mental health in Chinese adolescents. It is hypothesized that factors related to socio-economic situations, daily life, social support and school environment are associated with positive mental health among the sampled Chinese adolescents.

## Methods

### Sample

The study was performed in the urban area of the city of Weifang in Shandong Province of the eastern part of People’s Republic of China. By the year of 2016, the population of Weifang reached 9.35 million. The economic growth is steady in both industry and agriculture [49]. The socio-economic status of Weifang is considered to be high as the Shandong province is one of China’s most developed regions, with the third highest gross domestic product in 2014 of all Chinese provinces [50]. The total study population included students from grade 8 in twelve middle schools and grade 10 in five high schools in two urban districts of Weifang. Students in grade 8 from five middle schools were chosen by stratification by district and cluster sampling by school. As all middle school students were recruited according to their family address, we stratified the two urban districts in random sampling. While high school admitted students by their remarks in ‘high school entrance examination’, only cluster sampling was performed. A total of 5399 students, including 3044 students from grade 8 and 2355 students from grade 10, participated in the study. Among these students, the response rate was 100%. No one refused to respond to the questionnaire when the paper forms were distributed in the classroom, although 92% of respondents answered all 161 questions. In four schools, students were asked to fill in the questionnaire and return it in class after 1 h. In the three other schools, the questionnaires were completed at home and brought back to the teachers the following day. Students who were absent from school when the questionnaires were distributed were not included in the study.

### Measures

#### Questionnaire

This study was a part of a Sino-Swedish collaboration on positive mental health among adolescents, including MHC-SF. Sweden has a long history of student health services targeting the whole population and longitudinal measurement of adolescent health. The questionnaire used in this study was translated from a Swedish ongoing longitudinal survey known as "Survey of Adolescent Life in Vestmanland" (SALVe) [51]. The survey study was initiated in 1995 to investigate the health status and trends in health, lifestyle and school life in the whole county of Vestmanland, a region in mid Sweden. The MHC-SF and other items indicating general health, substance use, information technology exposure, school life and socio-economic situation were included in the 2014 version of SALVe questionnaire. Slight changes were made on several items in order to correspond better to a Chinese context. For example, ‘ice hockey’ was replaced by ‘table tennis’ in the items regarding sports. The questionnaire items were first translated into English by the last author and then into Chinese by the first author and other two Chinese researchers. Back translation into English was conducted by the first author to check the quality of translation with the last author. Therefore, the questionnaire in the cross-cultural study was considered to be appropriately adapted [52]. A pilot study on 385 students in Grade 8 was performed one month before the main study.

#### Mental health continuum-short form (MHC-SF)

The MHC-SF comprises 14 items, representing the three dimensions of well-being. A 6-point Likert scale is used to rate the feelings of the respondents in the past month (never, once or twice a month, about once a week, two or three times a week, almost every day, every day). A diagnosis of flourishing is made if the individuals feel 1 of the 3 hedonic well-being symptoms “every day” or “almost every day” and feel 6 of the 11 positive functioning symptoms “every day” or “almost every day” in the past month. A diagnosis of languishing is made if 1of the 3 hedonic well-being symptoms are perceived “never” or “once or twice a month” and 6 of the 11 positive functioning symptoms are perceived “never” or “once or twice a month”. Individuals who do not fit the diagnosis of “languishing” or “flourishing” mental health are categorized as “moderately mentally healthy” [53]. Evidence that the diagnosis is valid has been presented by Keyes in several publications [4, 14, 54] and the diagnosis used in a considerable number of papers since. The psychometric properties of MHC-Sf were evaluated on the same sample of Chinese adolescents [26]. Flourishing as a state where people have no depression, but high levels of well-being is the main focus of the research regarding positive mental health.

#### Variables

Socio-demographic variables used in the models included respondents’ gender, grade level, whether having a sibling, perceived family economy and family form. Perceived family economy was a single item that assesses a family’s current financial situations including 7 levels from low to high. Higher scores reflected perceptions of better family economic condition. In the analysis, this variable was then condensed into three levels: poor, moderate and good family economy. The variable ‘family form’ has two values: 1 for that adolescents live with both parents and 0 for that children live with one of the parents or other adults.

Self-satisfaction of weight/appearance were attained by a 5-point Likert item: ‘To what extent are you satisfied with your weight/appearance’ respectively. The variables of BMI, self-satisfaction of weight and self-satisfaction of appearance were grouped in three categories. Sleep quality, screen time, chronic stress, anxiety, depression, desire to learn and physical activity were dichotomized into two categories of ‘low’ and ‘high’. The behaviors of smoking, drinking and being bullied included two categories ranging from ‘never’ to ‘occasionally or often’. Parental support was measured by a single index of 3 items that reflect feelings of support for school life by parents (α = .81 for the subscale). Teacher’s support was measured by a single index of 4 items that reflect feelings of support for school life by teachers (α = .91 for the subscale). Social trust was measured by a single index of 3 items that reflect the attitude towards the society in a positive sense (α = .82 for the subscale). The percentage of missing data was no more than 5% for all relevant variables, except sleep quality (8.8%) and BMI (6.4%).

### Data analysis

The questionnaire data was analyzed using the statistical software SPSS 22. Prevalence was presented with proportions by categories of interest. Multivariate logistic regressions calculated Odds Ratios (OR) and 95% confidence intervals (CI) in analyzing the variables associated with ‘flourishing mental health’ as the dependent variable. Effect size measurements confirmed the significance between the variables in the model. The regression analysis was performed in two steps, in order to first create a crude model and then refine it into a final model. First, all potential indicator variables were checked for multicollinearity by performing Spearman correlation analysis (Spearman coefficient *ρ*_*S*_ > 0.70). The variables were screened in a multiple logistic regression analysis using the Enter method (the crude model). In the second step, the indicators with an estimate that was not significant in the crude model were dropped until all estimates in the model were statistically significant (the final model). The fit of the logistic models was assessed on the basis of the Hosmer-Lemeshow test. Nagelkerke Pseudo-*R*^2^ statistic was calculated to estimate the variance attributed to the predictors in a logistic regression model.

## Results

Table 2 presents the characteristics of the sampled Chinese adolescents. A total of 57.4% of the participants were flourishing. In grade 8, 57.9% of the boys and 60.6% of the girls were identified as flourishing. In grade 10, 54.1% of the boys and 55.0% of the girls were diagnosed as flourishing. Findings of the Chi-square tests indicated these differences in the prevalence of mental health in adolescents were significant.

**Table 2:** Selected sample characteristics

Characteristic	n^a^	%
Gender		
Female	2757	53.1
Male	2437	46.9
Grade		
8	3044	56.4
10	2355	43.6
Sibling		
0	4069	76.3
1 or more	1263	23.7
Family form		
Living with both parents	4595	87.0
Others	687	13.0
Perceived family economy		
Poor	843	16.2
Moderate	2212	42.5
Wealthy	2144	41.2
Collection method		
Answering at school	2756	51.0
Answering at home	2643	49.0
BMI		
Thinness	1719	34.0
Normal	2808	55.6
Overweight/obesity	526	10.4
Satisfaction of self-weight		
Dissatisfied	1361	25.2
Moderate	1413	26.2
Satisfied	2619	48.6
Satisfaction of self-appearance		
Dissatisfied	558	10.4
Moderate	1963	36.4
Satisfied	2868	53.2
Sleep quality		
Poor	2227	45.2
Good	2695	54.8
Physical activity		
Low level	2531	48.2
High level	2716	51.8
Stress		
Never or rare	4437	85.5
Often	751	14.5
Total screen time		
Up to 6 h per day	5148	95.4
6.5 or more hours per day	251	4.6
Smoking habit		
Never smoke	4906	93.0
Smoking or used to smoke	367	7.0
Drinking alcohol		
Never	3894	73.7
Occasionally or often	1393	26.3
Social trust		
Low	2900	56.8
High	2208	43.2
Desire to learn		
Weak	2272	43.8
Strong or moderate	2911	56.2
Teacher’s support		
Low	1896	37.0
High	3222	63.0
Parents’ support		
Low	2742	53.3
High	2406	46.7
Bullied at school		
Never	3001	58.2
Occasionally or often	2156	41.8
Total	5399	100

Note. ^a^Numbers vary due to missing data

Table 3 presents the effects of our chosen presumptive indicators of positive mental health in the crude model. The results of the multivariate logistic regression analysis showed that gender, perceived family economy, sibling, satisfaction of self-appearance, physical activity, sleep quality, stress, social trust, desire to learn, teacher’s support, parental support and being bullied at school were significantly associated with positive mental health. The Hosmer-Lemeshow *p*-value for the crude model (0.54) was substantially above 0.05. Accordingly, the multivariate logistic regression analysis was performed again after non-significant indicators were dropped from the model. The results show that total screen time was not significantly associated with positive mental health (*p* = 0.10). With this indicator removed from the model, the multivariate logistic model was tested and proved to be the final model with all indicators significantly associated with positive mental health. The Hosmer-Lemeshow *p*-value for the final model (0.45) was above 0.05 indicating adequate model fit.

**Table 3:** Indicators associated with positive mental health by multivariate logistic regression (crude model)

Variables^a^		OR^b^	95% CI
Gender	Boys	1.00	
	Girl	1.28	1.09–1.50
Grade	8	1.00	
	10	1.40	0.97–2.02
Collection method	Answering at home	1.00	
	Answering at school	1.32	0.92–1.89
Perceived family economy	Poor	1.00	
	Moderate	1.23	1.01–1.51
	Good	1.62	1.31–2.00
Sibling	0	1.00	
	1 or more	1.30	1.09–1.55
Family form	Living with both parents	1.00	
	Others	1.05	0.85–1.30
BMI	Overweight/obesity	1.00	
	Normal	1.07	0.81–1.40
	Thinness	0.98	0.77–1.26
Satisfaction of self-weight	Dissatisfied	1.00	
	Moderate	0.89	0.72–1.09
	Satisfied	1.01	0.82–1.26
Satisfaction of self-appearance	Dissatisfied	1.00	
	Moderate	1.55	1.19–2.03
	Satisfied	2.82	2.13–3.73
Physical activity	Low level	1.00	
	High level	1.31	1.13–1.52
Sleep quality	Poor	1.00	
	Good	1.89	1.61–2.21
Stress	Often	1.00	
	Never or rare	1.37	1.10–1.69
Total screen time	6.5 or more hours per day	1.00	
	Up to 6 h per day	1.47	1.04–2.08
Smoking habit	Smoking or used to smoke	1.00	
	Never	1.09	0.81–1.48
Drinking alcohol	Occasionally or often	1.00	
	Never	1.06	0.89–1.26
Social trust	Low	1.00	
	High	2.67	2.28–3.12
Desire to learn	Weak	1.00	
	Strong or moderate	1.44	1.24–1.68
Teacher’s support	Low	1.00	
	High	1.42	1.22–1.65
Parents’ support	Low	1.00	
	High	1.71	1.46–2.00
Bullied at school	Occasionally or often	1.00	
	Never	1.43	1.23–1.66

Note. *OR* Odds ratio, *CI* Confidence interval

^a^Outcome variables were dichotomized in symptomatic and non-symptomatic categories according to literature

^b^Each category presented is tested against the reference category (OR = 1.00)

Table 4 presents the final model with odds ratios and 95% confidence intervals for all significant indicators with regards to positive mental health of young adolescents. The strongest effect in the model was seen for satisfaction of self-appearance with an OR of 2.75. The final model explained 31% of the variance in positive mental health (Nagelkerke pseudo-*R*^2^ = 0.310).

**Table 4:** Final model of indicators associated with positive mental health by multivariate logistic regression

Variables^a^		OR^b^	95% CI
Gender	Boys	1.00	
	Girl	1.31	1.13–1.52
Perceived family economy	Poor	1.00	
	Moderate	1.23	1.01–1.50
	Good	1.63	1.33–2.00
Sibling	0	1.00	
	1 or more	1.32	1.12–1.56
Satisfaction of self-appearance	Dissatisfied	1.00	
	Moderate	1.46	1.14–1.86
	Satisfied	2.75	2.15–3.51
Physical activity	Low level	1.00	
	High level	1.33	1.16–1.54
Sleep quality	Poor	1.00	
	Good	1.90	1.64–2.20
Stress	Often	1.00	
	Never or rare	1.41	1.14–1.73
Social trust	Low	1.00	
	High	2.53	2.18–2.93
Desire to learn	Weak	1.00	
	Strong or moderate	1.48	1.28–1.72
Teacher’s support	Low	1.00	
	High	1.43	1.23–1.66
Parents’ support	Low	1.00	
	High	1.73	1.49–2.00
Bullied at school	Occasionally or often	1.00	
	Never	1.44	1.25–1.66

Note. *OR* Odds ratio, *CI* Confidence interval

^a^Outcome variables were dichotomized in symptomatic and non-symptomatic categories according to literature

^b^Each category presented is tested against the reference category (OR = 1.00)

## Discussion

This is, to the best of our knowledge, the first study to comprehensively assess the prevalence of positive mental health and its correlates in Chinese adolescents. More than half (57.4%) of the participants were diagnosed as flourishing. The variables significantly associated with positive mental health in the regression models included gender, perceived family economy, the occurrence of sibling(s), satisfaction of self-appearance, physical activity, sleep quality, stress, social trust, desire to learn, support from teachers and parents as well as whether being bullied at school.

The findings of the present study revealed a high prevalence of positive mental health for adolescents compared to the previous studies using MHC-SF to assess positive mental health (see Table 1), where the prevalence in most countries ranged between 10 and 30% [14, 16–20] and reached 43.4% in Chinese adults [25]. Hypothetical explanations for the high prevalence of positive mental health in this study might be the high socio-economic status of Weifang as well as the younger age of the participants compared to other similar studies. Keyes suggested that the prevalence of flourishing decrease as age increase during adolescence [41]. Also, younger adolescents reported higher prevalence of flourishing than older adolescents in an Indian study [21]. A study among Swedish adolescents and adults between 16 and 29 years found that positive mental health decreased with age [32]. Our findings suggest that the prevalence of flourishing in Chinese adolescents was higher than most of other countries. However, this is still in need of comprehensive investigation, especially from a cultural dimension. China is described as a collectivist society with a high degree of power distance [55, 56]. This means that group norms dominate over individual wishes and that superiors, such as teachers and parents, are respected and obeyed. It is important to have a sense of belonging to groups, especially to the family [55, 57]. For example, researchers found fewer behavior problems in classrooms and schools in which students highly respect their teachers and value self-discipline in China, and related that to the Confucian values [58]. The inherent respect for superiors might be the explanation to the very high respondent rate of the questionnaire, as teachers requested students to respond. Support from family and teachers were also significantly associated with positive mental health in our study. Another explanation may be the positive attitudes of Chinese people towards the economic development of their country. According to Pew Research Center, 90% of Chinese rated the economic conditions of their country as good, which was the highest rating among all the 40 countries in the survey [59]. Also, 88% of the Chinese respondents believed that when today’s children grow up they will be better off financially than their parents. Another survey showed that the Chinese public was optimistic about the long-term economic future, “in particular, their positive outlook stands in stark contrast to the pessimism found in the United States and much of Europe” [60]. We found that social trust strongly indicates positive mental health in the current study (OR = 2.53, 95% CI = 2.18–2.93), which supports this hypothesis.

In our study, girls showed slightly better positive mental health than boys (see Table 4). Most previous studies using MHC-SF reviewed in this paper do not show any significant difference in positive mental health between males and females [15, 17, 25, 34, 61]. As this is the first study assessing positive mental health of Chinese adolescents using MHC-SF, we are unable to compare our results with previous studies in China. In terms of psychiatric disorders, however, some researchers found a higher prevalence among Chinese boys than among girls [62]. Girls were found to be more flourishing than boys in India [21], indicating that there might be characteristics shared in Asian countries regarding positive mental health. One hypothetical explanation of the slightly higher prevalence of positive mental health among Chinese girls in our study could be that they perceive a better relationship to their parents than boys [63].

The indicators that were found to have the highest impact on positive mental health of Chinese adolescents were social trust, satisfaction of self-appearance, sleep quality, parents’ support and perceived family economy. These are indicators on the individual and family level, except for social trust, which is an indicator on a community level. Our findings were in accordance with the determinant indicators of Orpana et al., of family relationships and parenting style, and social support and networks [5], as well as Maher and Waters’ individual determinant of a strong sense of self-worth [44].

Perceived family economy was identified as a significant indicator of positive mental health. In the current study, only one item assessed perceived family economy: ‘Imaging society as a ladder. If you think about your family’s finance in comparison with the wider community, where would you place your family on the scale below?’There was no objective data of family income because no questionnaire for parents was distributed. Instead, young participants described the impression of their parental income. We emphasized that the family economy was perceived by the participants because they were rating the economy of someone else other than themselves. In a meta-analysis of subjective socio-economic status and adolescent health, positive associations were found between socio-economic status including family economy and better health outcomes in adolescence [64]. Previous studies found evidence for that socio-economic status was related to gender, income, education and employment as determinants of positive mental health in United States, European, Australian and Asian countries including China [4, 15, 27–33]. Adolescents with one or more siblings (23.7% of the respondents) reported a higher level of positive mental health than those who were the only child of their parents, which is in line with previous findings [65]. Sibling relationship is considered to have great influence in adolescence and may affect positive mental health [65]. In China, the family planning program has experienced several transitions and varied in different areas and time periods since the 1970s. As a result, the rigorous one-child policy was modified in the 1980s so that the families meeting certain criteria were allowed to have more than one child, usually in rural areas [66]. Thus, a proportion of adolescents with one or more siblings were identified in this study.

Body image satisfaction is often defined as the degree to which individuals are satisfied with their physical appearance, especially weight and shape [67]. It is noteworthy that BMI and satisfaction of self-weight were not significantly associated with positive mental health in the final regression model. Instead, the satisfaction of self-appearance was found to be an evident predictor of positive mental health in Chinese adolescents. Our findings concerning BMI were in line with a previous study on Chinese college students [35]. Also, body dissatisfaction was found to be negatively associated with quality of life among adolescents regardless of gender [68], which strongly supports our results. Based on this, the authors hypothesize that satisfaction of self-appearance influences positive mental health. However, positive mental health can also cause satisfaction of self-appearance and self-satisfaction of weight, and a third variable influencing both positive mental health and satisfaction of self-appearance/self-weight, for example, mental disorder (i.e., eating disorder). Unfortunately, our data collection and analysis cannot specify the causal relationships between these variables.

There was no association identified between smoking habits and positive mental health. Only 7.0% of the participants in our study reported current or previous smoking. However, the prevalence of smoking in Chinese adolescents is generally high [69]. Considering that smoking among males is a severe public health problem in China [70], the relatively low prevalence of smoking among the adolescents included in the study was surprising. As the relationship between smoking and low socio-economic status is strong [71], the low prevalence of smoking might be explained by the relatively high socio-economic status of Weifang. Moreover, Davoren et al. found that smoking was not associated with mental health and well-being among students in Ireland and assumed that the smoking habits were underreported (27% for men and 25.6% for women) [34]. This might also be the case in our study.

A strength of this study is that the sample size is larger than the previous studies utilizing MHC-SF listed in Table 1 [14, 16–21, 25, 61], except for the Canadian sample which represented the national population [15]. We also included a comprehensive range of potential explanatory variables from multiple aspects of adolescent life. After successfully validating the MHC-SF in Chinese adolescents [26], we found that Keyes’ definition of positive mental health fitted well into the Chinese context and thus examined the prevalence of positive mental health and associations with a range of indicators of positive mental health. There are also limitations of the study. First, the study was performed in an economically developed area of China. Thus, the family income might be higher among our respondents than in other less developed areas considering the regional disparities in China [72]. Second, the extreme dependence on self-reported information might lead to a recall bias affecting the results. For example, respondents might not remember or avoid to give responses that are perceived to be socially undesirable [73], especially in adolescents [74]. In order to reduce social pressure and possible social desirability effects, the self-administered questionnaire was answered anonymously [75]. The cross-sectional study design did not allow further assumptions on the causality relationship between positive mental health and potential risk and protective factors. Finally, questionnaires were answered in two different ways depending on the choice of the school headmasters. In four out of the seven schools, students completed the questionnaire in the classrooms in during a one-hour period. Students in the three other schools filled in the forms at home and returned them to school teachers on the following day. It should be noted that all students from grade 10 completed the questionnaires at home whereas most students from grade 8 responded to the questions at school. However, the variable ‘collection method’ was not significantly associated with positive mental health in the crude model, indicating that the different circumstances under which the questionnaire was answered had no effect on positive mental health.

## Conclusion

Our study adds to the knowledge of prevalence of positive mental health in Chinese adolescents measured by the MHC-SF, as well as indicators of positive mental health. The prevalence of positive mental health among the sampled Chinese adolescents was considerably higher than reported in most of the previous studies using MHC-SF. Unlike most other studies of this kind, girls reported a higher level of positive mental health than boys. The indicators of social trust, satisfaction of self-appearance, sleep quality, parents’ support and perceived family economy were associated with a high level of positive mental health. Our study was performed in one specific city of China, Weifang, with a high socio-economic status. Thus, further studies are required to assess the positive mental health of adolescents in other geographical areas of China and in areas of different socio-economic status. Future longitudinal studies should focus on investigating relevant causality and potential bidirectional associations. To improve positive mental health among Chinese adolescents, policy-makers should focus on strategies and actions supporting social trust and support as well as acknowledging the support and socio-economic status of families as significant factors influencing positive mental health. The measurement of positive mental health should be used to identify the vulnerable groups that could benefit from intervention and assess the baseline mental health status of those groups. Our findings of risk and protective factors contribute to the mental health strategy in public health actions.

## Abbreviations

CI: Confidence interval
MHC-SF: Mental Health Continuum-Short Form
OR: Odds ratio
SALVe: Survey of Adolescent Life in Vestmanland
